# Pyonephrosis and urosepsis in a 41-year old patient with spina bifida: Case report of a preventable death

**DOI:** 10.1186/1754-9493-6-10

**Published:** 2012-05-21

**Authors:** Subramanian Vaidyanathan, Fahed Selmi, Bakul Soni, Peter Hughes, Gurpreet Singh, Kamesh Pulya, Tun Oo

**Affiliations:** 1 Regional Spinal Injuries Centre, Southport and Formby District General Hospital, Town Lane, Southport, PR8 6PN, UK; 2 Department of Radiology, Southport and Formby District General Hospital, Town Lane, Southport, PR8 6PN, UK; 3 Department of Urology, Southport and Formby District General Hospital, Town Lane, Southport, PR8 6PN, UK; 4 Department of Cardiology, Southport and Formby District General Hospital, Town Lane, Southport, PR8 6PN, UK

## Abstract

****Background**:**

Urological complications are the major cause of ill health in patients with spina bifida. Urinary sepsis accounted for the majority of admissions in patients with spina bifida. As the patient grows older, changes occur in the adult bladder, leading to increases in storage pressure and consequent risk of deterioration of renal function, which may occur insidiously.

****Case presentation**:**

A 34-year-old male spinal bifida patient had been managing neuropathic bladder by penile sheath. Intravenous urography revealed normal kidneys. This patient was advised intermittent catheterisations. But self-catheterisation was not possible because of long, overhanging prepuce and marked spinal curvature. This patient developed repeated urine infections. Five years later, ultrasound examination of urinary tract revealed hydronephrotic right kidney with echogenic debris within the collecting system. There was no evidence of dilatation of the ureter near the vesicoureteric junction. The left kidney appeared normal. There was no evidence of calculus disease seen in either kidney. Indwelling urethral catheter drainage was established.

Two years later, MAG-3 renogram revealed normal uptake and excretion by left kidney. The right kidney showed little functioning tissue. Following a routine change of urethral catheter this patient became unwell. Ultrasound examination revealed hydronephrotic right kidney containing thick hyper-echoic internal septations and debris in the right renal pelvis suspicious of pyonephrosis. Under both ultrasound and fluoroscopic guidance, an 8 French pig tail catheter was inserted into the right renal collecting system. 150 ml of turbid urine was aspirated immediately. This patient developed large left pleural effusion, collapse/consolidation of the left lower lobe, a large fluid collection in the abdomen extending into the pelvis and expired twenty days later because of sepsis and respiratory failure.

****Conclusion**:**

Although penile sheath drainage may be convenient for a spina bifida patient and the carers, hydronephrosis can occur insidiously. With recurrent urine infections, hydronephrotic kidney can become pyonephrosis, which is life-threatening. Therefore, every effort should be made to carry out intermittent catheterisations along with antimuscarinic drug therapy.

## **Introduction**

Spina bifida, the most frequent permanently debilitating birth defect, results in major urological problems of voluntary bladder control and bowel function, which may impair quality of life [1]. Renal damage and renal failure are among the most severe complications of spina bifida [2]. Therefore, patients with spina bifida require longitudinal urological care as they transition from childhood to adolescence and then to adulthood. Issues important to urological health, such as protection of the upper tracts and prevention of incontinence, need vigilant follow-up throughout the patient's life [3].

Cahill and Kiely from Department of Urology, Cork University Hospital, Ireland found that urological complications were the major cause of ill health during childhood and adult life of patients with spina bifida. Urinary sepsis accounted for the majority of admissions (62 %) in patients with spina bifida currently attending a specialised multidisciplinary clinic over a period of six months [4].

In spina bifida patients, the primary objective is protecting kidney function by establishing a good capacity, low-pressure urinary reservoir. Ensuring adequate bladder and bowel continence is also paramount for enhancing self-esteem and independence. Medical therapy incorporating clean intermittent catheterization and antimuscarinic medication is the cornerstone of neurogenic bladder management and often the only intervention required to achieve the above goals [5]. Although construction of a continent urinary reservoir is practised widely, MacNeily and associates [6] did not note an improvement in overall quality of life following reconstruction of lower urinary tract in spina bifida cases. Correcting only one system in a profound multisystem disability may be insufficient to improve health related quality of life or perhaps only caregiver quality of life is improved.

As the patient grows older, changes occur in the adult bladder, leading to increases in storage pressure. Medical and surgical management should aim to preserve renal function as well as the maintenance of continence in the face of the growing and changing urinary tract. Otherwise, renal function may begin or continue to deteriorate and renal disease may become the leading cause of adult death [7].

We present an adult male patient with spina bifida, who had been managing neuropathic bladder by penile sheath and developed hydronephrosis. Then the method of bladder drainage was changed to indwelling urethral catheter. This patient developed repeated urine infections. The hydronephrotic kidney became pyonephrosis and the patient succumbed to overwhelming sepsis.

## **Case presentation**

**Figure 1:**
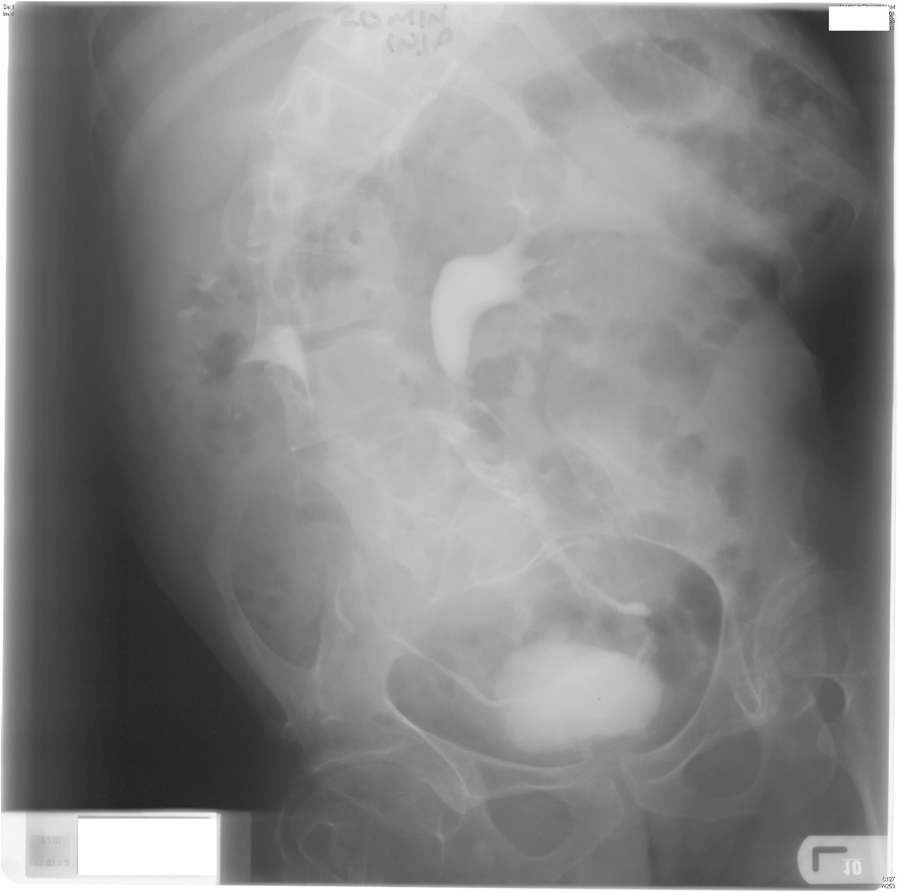
Intravenous urography performed when the patient was 31 years old: no radio opaque calculi, normal kidneys and ureters; trabeculated, small capacity urinary bladder.

A male patient, born in 1970 with congenital neural tube defect and thoracic paraplegia, had been managing his bladder with penile sheath. When this patient was thirty years old, he developed an ulcer all around the circumference of base of penis. A tape had been applied tightly in a circumferential manner to secure penile sheath. Tight application of penile sheath led to ulceration of skin and subcutaneous tissue as well as oedema of distal shaft of penis. An indwelling urethral catheter was inserted. Suprapubic cystostomy was considered but the patient and his parents were not happy about it. Intravenous urography revealed no renal tract calcification; normal kidneys, ureters and urinary bladder. Intravenous urography, performed a year later, revealed no radio opaque calculi, normal kidneys and ureters, and trabeculated, small capacity urinary bladder (Figure 1). This patient was advised to manage his bladder by penile sheath drainage and twice a day intermittent catheterisation along with antimuscarinic drug. Self-catheterisation was not possible because of very long overhanging prepuce and marked spinal curvature. Therefore, the patient continued to drain his bladder through a penile sheath.

When this patient was 35 years old, intravenous urography revealed normal kidneys, ureters and bladder. His penis became swollen due to tight application of penile sheath. Therefore, indwelling urethral catheter drainage was advised. In the community, a health professional inserted a female Foley catheter and inflated the balloon of female Foley catheter in the bulbous urethra; consequently, there was profuse bleeding from urethra. A male Foley catheter was inserted per urethra. Six weeks later, the catheter was removed and size 40 Clear Advantage sheath was applied over the penis. Proximal rim of the sheath was cut at two places so that there would not be any circumferential constriction upon the penis.

When this patient was 38 years old, this patient developed urine infection and received antibiotic from his doctor. A week later, his father noticed swelling of left testis; the patient however did not notice the swollen testis. He had been managing his bladder with penile sheath, which was changed once in two days. This patient was prescribed Ciprofloxacin 500 mg twice a day for two weeks. Ultrasound scan showed enlarged left testis with increased blood flow suggestive of orchitis. The right testis appeared relatively well preserved.

**Figure 2:**
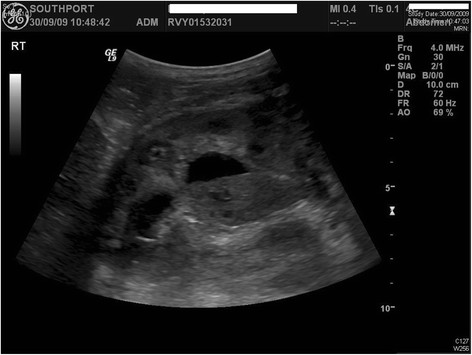
**Ultrasound examination of urinary tract, performed when the patient was 39 years old: the right kidney was hydronephrotic with dilated renal pelvis**. There was echogenic debris within the right collecting system.

**Figure 3:**
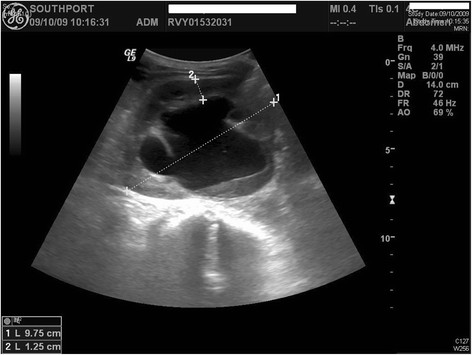
**Ultrasound scan of urinary tract repeated ten days later after the patient had received antibiotic: there was less debris within the hydronephrotic right kidney than at the previous scan**. There was however still significant hydronephrosis with antero-posterior diameter of right renal pelvis being 5.7 cm.

A year later, this patient developed repeated urine infections and urine was cloudy. This patient had received three courses of antibiotics from his doctor: two lots of Cephalexin, and then Ciprofloxacin. This patient did not have rigors but had been having night sweats. Sweating occurred most nights since the beginning of urine infection. Ultrasound examination of urinary tract revealed that the right kidney was hydronephrotic with dilated renal pelvis. There was echogenic debris within the right collecting system (Figure 2). There was no evidence of dilatation of the ureter near the vesicoureteric junction. The left kidney appeared normal. There was no evidence of calculus disease seen in either kidney. Urinary bladder appeared normal. Microbiology of urine showed growth of Pseudomonas aeruginosa and Enterococcus faecalis. Penile sheath was removed and urethral catheter was inserted. Ultrasound scan of urinary tract was performed after ten days, which showed less debris within the hydronephrotic right kidney than at the previous scan. There was however still significant hydronephrosis with antero-posterior diameter of right renal pelvis being 5.7 cm (Figure 3). The right kidney measured 9.7 cm in length with 1.25 cm cortical thickness. There was still little debris in the right collecting system. Left kidney was not hydronephrotic. Bladder was catheterised. 

The patient, his father, District Nurse and the General Practitioner were requested to consider intermittent catheterisations instead of long-term indwelling catheter drainage. Likely complications of long-term indwelling urinary catheter drainage such as urine infections, stones in urinary bladder were explained to the patient and health professionals. Self-catheterisation was impossible because of marked spinal curvature and long, overhanging prepuce. This patient’s life style involved going out and facilities for urethral catheterisation were not available in many public places. Therefore, intermittent catheterisation regime could not be implemented. The patient had urethral catheter, which was changed every four weeks in spinal injuries unit. This patient did not receive any antibiotic after routine change of urethral catheter.

When this patient was forty one years old, ultrasound examination revealed marked right hydronephrosis. In comparison to the previous ultrasound examination, there was increased dilatation of the renal pelvis and thinning of renal cortex. There was layering of debris within the dilated renal pelvis. The left kidney was normal. MAG-3 renogram showed single functioning left kidney demonstrating normal uptake and excretion. Right kidney showed little functioning tissue.

Following a routine change of urethral catheter, this patient became unwell. This patient attended emergency department thirty-six hours after change of urethral catheter with symptoms of mild abdominal pain, lethargy, diminished appetite, and decreased urine output. On examination, temperature was 34 degrees Celsius; Heart rate: 107; Respiratory rate: 18 per minute; Blood pressure: 85/55 mm Hg; Oxygen saturation: 100 %. Abdomen was distended. Skin over right flank was looking red. Based on previous microbiology report of urine culture, which had shown growth of Pseudomonas aeruginosa sensitive to Tazocin, this patient was prescribed Tazocin.

**Figure 4:**
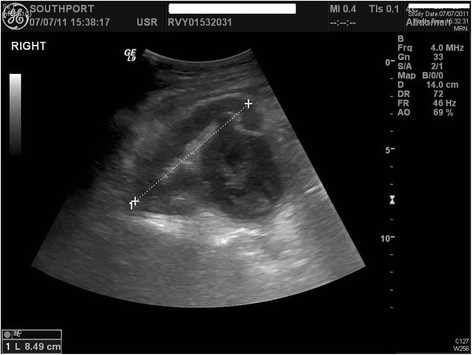
**Ultrasound examination of urinary tract performed when the patient was 41 years old: moderate hydronephrosis of right kidney was seen**. Right renal pelvis was grossly dilated with thick internal echogenic septations and echogenic debris suggestive of pyonephrosis.

Ultrasound examination of urinary tract revealed normal echotexture of left kidney, which measured 10.5 cm with good cortico-medullary differentiation and cortical depth. There was no left hydronephrosis. Right kidney measured 8.4 cm with moderate hydronephrosis. Right renal pelvis was grossly dilated with thick internal echogenic septations and echogenic debris (Figure 4). Free fluid was noted in the abdomen and pelvis. Although grossly dilated right renal pelvis was a long-standing finding, thick heper-echoic internal septations and debris in the right renal pelvis was suspicious of pyonephrosis.

Microbiology of urine revealed growth of multi-drug resistant coliforms, sensitive toMeropenem. Therefore, Meropenem was prescribed after discontinuing Tazocin.

Under both ultrasound and fluoroscopic guidance, an 8 French pig tail catheter was inserted into the right renal collecting system. 150 ml of turbid urine was aspirated immediately.

Computed Tomography of chest, abdomen and pelvis revealed large left pleural effusion, collapse/consolidation of the left lower lobe, collapse/consolidation of the posterobasal segments of the left upper lobe and minor right basal atelectasis. A large fluid collection was noted in the abdomen extending into the pelvis with enhancing walls. Fluid was also noted around the spleen, in the paracolic gutters and in the root of the mesentery. Liver, spleen, pancreas, gallbladder, adrenals and the left kidney appeared normal.

Ultrasound guided drainage of intra-abdominal collection was performed. A 12 French self-locking pigtail catheter was inserted into the abdominal collection, which looked predominantly clear fluid but with occasional internal septa.

**Figure 5:**
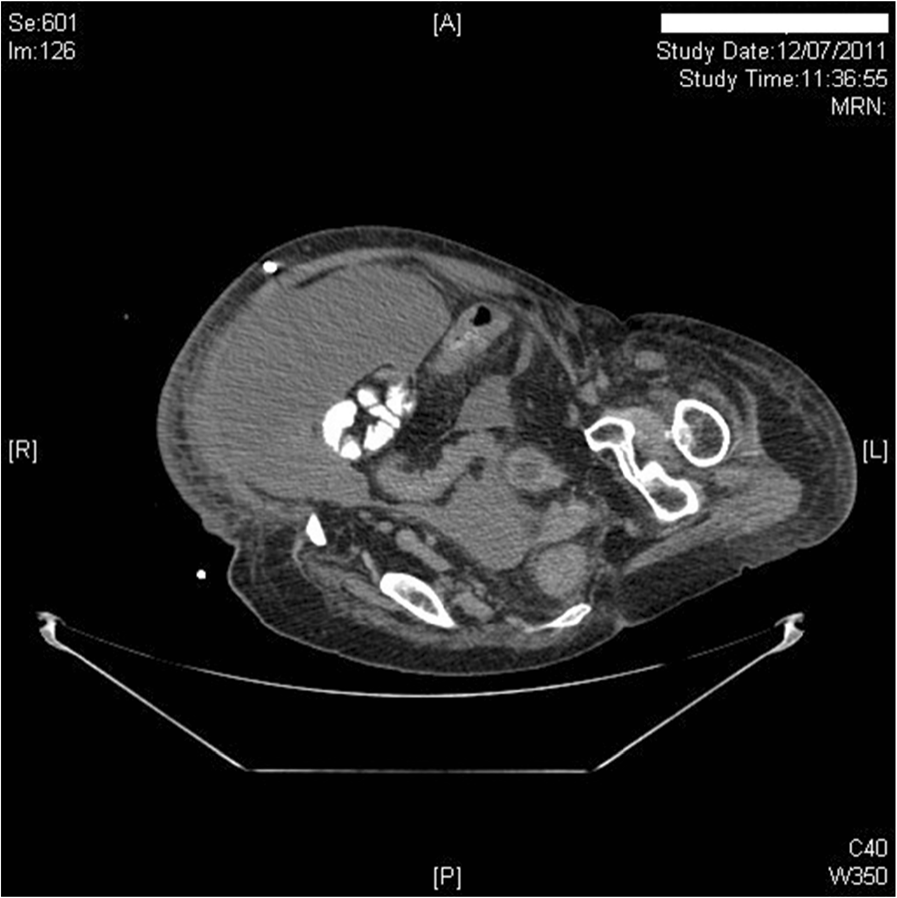
**Computed Tomography of chest, abdomen and pelvis, performed after percutaneous drainage of right pyonephrosis: a large fluid collection was seen in the abdomen extending into the pelvis with enhancing walls**. Fluid was also noted around the spleen, in the paracolic gutters and in the root of the mesentery.

Computed tomography revealed a significant left sided pleural effusion with a left subphrenic fluid collection measuring four cm in depth. There was distension of the stomach and small bowel loops. Nephrostomy tube was in situ in right kidney, which was decompressed. There was still a significant lower abdominal fluid collection (Figure 5). Pigtail catheter was in situ within the collection. Limited drainage was possibly due to septa seen on ultrasound. Therefore, under ultrasound guidance, re-positioning of the drainage tube into the upper component of the multi-locular pelvic inflammatory collection was performed, followed by aspiration of 135 ml of clear yellowish fluid. The following day, under ultrasound guidance, an 8 French pigtail catheter was introduced into the caudal portion of the multi-loculated pelvic inflammatory collection. The distal end of the drainage tube was positioned in the most dependent point of the collection. Immediately, 90 ml of clear yellowish fluid was aspirated. The fluid was found gelatinous and changed partially into solid state after aspiration, a finding suggestive of high protein contents. The drain tubes were flushed every six hours using 20 to 40 ml of 0.9 % sodium chloride.

Chest X-ray revealed large left basal effusion. A chest drain was inserted. Chest X-ray, taken on the following day, showed left chest drain in situ, extensive left basal consolidation, and large left basal effusion. Right lung was clear. The next day, chest X-ray revealed the drain in situ with mild left surgical emphysema and increasing consolidation of left lung.

Computed tomography, performed two days later showed an increase in size of the left sided pleural effusion since the last examination. There was secondary collapse of the left lower lobe. Mild right sided pleural effusion was present along with right basal atelectasis posteriorly. There was some air and fluid in the posterior chest wall muscles medial to the scapula. Marked gaseous distension of proximal and mid small bowel loops was evident. There was mild decrease in size of the fluid collection in the right side of the abdomen which tracked across the mid line anteriorly; drains were present within this collection. Right nephrostomy was in situ.

Using Kimmel needle, access to the left pleural effusion was established in the upper mid axillary region. Three mls of thick clear fluid was aspirated; however, there was no free aspirate; hence drain was not inserted. Patient’s condition deteriorated despite intravenous antibiotic therapy, parenteral nutrition and chest physiotherapy. Arterial blood gas showed: pH: 7.218; pCO2: 13.80 kPa; pO2: 11.74 kPa; Actual bicarbonate: 41.2 mmol/L; Standard Bicarbonate: 32.5 mmol/L; Base Excess: 8.8 mmol/L. This patient succumbed to respiratory failure. Post-mortem examination was not performed.

## **Discussion**

Persons with spina bifida have hospitalisations that are beyond what the general population experiences. These conditions may be potentially preventable with appropriate ambulatory care. Wilson and associates [8] observed that persons with spina bifida also had a greater risk for readmission within 30 days of discharge from their last hospitalisation.

Urological complications are the major cause of ill health during childhood and adult life of patients with spina bifida but the significance of urinary tract disease on the individual and the healthcare services is under-emphasised. Dysfunctional bladder outlet causes febrile urinary tract infections and subsequent renal scarring. The development of secondary vesicoureteric reflux increases the risk of renal scarring and chronic kidney disease. Intermittent catheterisations with antimuscarinic drugs (based on the findings of urodynamics) are recommended to prevent renal complications. Overnight catheter drainage, Botox, and eventually augmentation cystoplasty may be required for poorly compliant bladders [4].

Ahmad and Granitsiotis [7] state that despite not being validated in the follow-up of adult spina bifida patients, serum creatinine, ultrasound and urodynamics should be performed annually and these tests represent currently the best tools available. To protect the upper urinary tract in patients with spina bifida, Dik and associates [9] recommend starting children early on clean intermittent catheterisation, which is the preferred treatment, and prescribing anti-muscarinic agents to counteract detrusor instability. Intermittent catheterisation and anti-muscarinic therapy will ensure low intravesical pressure. Such proactive treatment of risks for upper urinary tract deterioration results in a negligible loss of renal function.

However, efforts to implement clinical guidelines are not always successful. This case illustrates the wide gap which exists between knowledge on management of neuropathic bladder and actual care of a spina bifida patient. Such a wide gap between knowledge (annual ultrasound, urodynamics, measurement of serum creatinine, and implementation of intermittent catheterisation with anti-muscarinic drug therapy) and actual clinical practice exists not only in relation to management of neuropathic bladder but also in the treatment of other urological diseases. Gravas and associates [10] found that the difficulties in translation of benign prostatic hyperplasia guidelines into clinical practice were related to (1) lack of knowledge, (2) differences in routine practices, (3) beliefs, (4) cost, (5) availability, and (6) reimbursement policy. Bridging such a wide gap in the implementation of clinical guidelines on management of spina bifida patient represents a challenging task for doctors and health care managers.

At the age of 34 years, intravenous urography showed normal kidneys, ureters and bladder in this patient. But after five years, ultrasound examination revealed hydronephrosis of right kidney while this patient’s bladder was being drained by a penile sheath. For nearly five years between 2004 and 2009, this patient did not undergo assessment of urinary tract. Had we implemented intermittent catheterisation along with adequate anti-muscarinic therapy, it was likely that renal function would have been preserved in this patient. This case is a poignant reminder of the existence of difficulties in translation of knowledge into clinical practice. In this instance, we were unable to implement intermittent catheterisation and anti-muscarinic therapy in a spina bifida patient. Further, this case shows the need to send a reminder by post, telephone call, e-mail or a text message to the mobile phone requesting spina bifida patients to come to spinal unit for global follow-up including assessment of urinary tract.

When hydronephrosis was discovered, this patient was advised intermittent catheterisations along with antimuscarinic therapy. But self-catheterisation was not possible because this patient had marked spinal curvature and a long, overhanging prepuce. The patient’s life style included going out and facilities for intermittent catheterisation were not available in many public places. Therefore, it was not possible to implement intermittent catheterisations and the patient was managed by urethral catheter drainage. Compared with other forms of bladder management, use of an indwelling catheter, is associated with more pressure ulcers and longer and more hospitalisations for all causes and urology-specific causes [11].

Snodgrass and Gargollo [12] recommend that persons with spina bifida who have urodynamic evidence of uninhibited contractions or rising pressure during filling should be started on anticholinergics and clean intermittent catheterisation, or have their dosage increased until pressures less than 40 cm H_2_O and detrusor areflexia are achieved. Augmentation cystoplasty is indicated in patients with hydronephrosis or vesico-ureteric reflux, and end-filling pressures or detrusor leak point pressure > 40 cm H_2_O despite anticholinergic therapy to the point of patient tolerance. Kokorowski and associates [13] state that augmentation cystoplasty is the mainstay of surgical treatment for *medically refractory* neurogenic bladder in patients with spina bifida.

However, it should be remembered that patients augmented with ileal or colonic segment for a congenital bladder anomaly have a 7–8 fold and gastric augments a 14–15 fold increased risk for the development of bladder cancer over standard norms. The incidence of cancer developing per decade following surgery was 1.5 % for ileal or colonic augmentations and 2.8 % for gastric bladder augmentations. The majority of cancers developing within the augmented bladder are at advanced stages at the time of diagnosis [14]. Urothelial carcinomas, which developed after augmentation cystoplasty were extremely aggressive and exhibited distinct morphological, immunohistochemical and genetic characteristics. In the morphological evaluation, all tumours were high-grade (grade 3) invasive urothelial carcinoma comprising various architectural patterns with brisk mitoses and tumour necrosis [15]. Kokorowski and associates showed that annual screening for malignancy among patients with spina bifida with cystoplasty using cystoscopy and cytology was unlikely to be cost effective at commonly accepted willingness to pay thresholds [13].

In order to implement intermittent catheterisation regime, this patient required carers, who could perform catheterisations both during day and night. Had facilities for carers been available, it might have been possible to discard the indwelling catheter and manage the bladder by intermittent catheterisations. This patient could have used a catheter with a bag already attached to the catheter for catheterisations in public places where suitable toilet facilities were not available (LoFric Hydro-Kit; manufactured by AstraTech Ltd, Stonehouse, Gloucestershire, GL10 3SX, United Kingdom).

In clinical practice, barriers to intermittent catheterisation are as follow:

1. Caregivers or nurses are not available to carry out five or six catheterisations a day.

2. Lack of time to perform intermittent catheterisations.

3. Unavailability of suitable toilet facilities in public places, including restaurants and offices.

4. Redundant prepuce in a male patient, which prevents ready access to urethral meatus.

5. Urethral false passage.

6. Urethral sphincter spasm requiring the use of flexible-tip catheters and álpha-adrenoceptor-blocking drugs.

7. Reluctance to perform intermittent catheterisation in patients >60 years by some health professionals.

8. Difficulty in accessing the urethral meatus for catheterisation while the patient is sitting up, especially in female patients [16].

This case demonstrates the urgent need for (1) trained caregivers who can perform intermittent catheterisation, and (2) public toilets with adequate space to accommodate a spina bifida patient, who uses electric wheel chair and a carer, who will perform intermittent catheterisation.

The ideal marker for measurement of renal function in persons with spina bifida is Cystatin-based e-GFR. Creatinine-based methods are insensitive because of low muscle mass and under-developed musculature in the legs [17]. Only Cystatin C-based e-GFR can reliably assess global renal function in these patients. However, unilateral renal damage requires nuclear medicine scans, such as 99mTc DMSA. In this patient, MAG-3 renogram showed little functioning tissue in right kidney. We did not have facilities to estimate Cystatin-based e-GFR.

The General Practice Research Database show that those spina bifida patients, who have survived to age 10 years still have double the mortality of the general population [18]. People with neural tube defects were found to have a substantially increased risk of renal failure compared with the general population [19]. Detrusor hyperreflexia with or without detrusor-sphincter dyssynergia and hypo- or acontractile detrusor undermine safe, effective and controlled storage and voiding of urine and predispose to reflux nephropathy. Therefore, patients with neural tube defects with lower urinary tract dysfunction would be expected to have increased risk of renal failure. This patient developed hydronephrosis of right kidney while the neuropathic bladder was drained by a penile sheath. Subsequently, the hydronephrotic kidney became pyonephrosis and the patient succumbed to sepsis.

This case raises some controversies in clinical management:

· Should hydronephrotic, non-functioning kidney be removed in spina bifida patients? What is the risk of nephrectomy and what is the risk of non-operative management of hydronephrotic kidney?

· Should prophylactic antibiotic be given after routine change of urinary catheter especially in those spina bifida patients, who have a non-functioning, hydronephrotic kidney?

· This patient developed capillary leak syndrome following severe sepsis originating from the urinary tract. The systemic capillary leak syndrome is an extremely rare disorder characterised by transient episodes of hypotensive shock and anasarca thought to arise from reversible microvascular barrier dysfunction [20]. Although the high prevalence of a monoclonal gammopathy of unknown significance in systemic capillary leak syndrome suggests a pathogenic contribution of endogenous immunoglobulins, the mechanisms of vascular hyperpermeability remain obscure. Lambert and associates [21] found intravenous immunoglobulins to be effective against systemic capillary leak syndrome symptoms in three patients, but their exact mechanism remained unknown. Would intravenously administered immuno-globulins have altered the course of events in this patient?

### **Take home message in non-medical language**

Penile sheath drainage may be convenient for a spina bifida patient and the carers. But, dilatation of the upper urinary tract with progressive deterioration of kidney function can occur insidiously. Urine infections predispose to formation of pus in a dilated kidney. Such complications can be life-threatening. Therefore, every effort should be made to carry out intermittent catheterisations in persons with spinal cord injury or spinal bifida. Along with intermittent catheterisation, medicines should be taken to reduce bladder spasms and decrease pressure within the urinary bladder.

## **Conclusion**

Penile sheath drainage or indwelling urinary catheter may be convenient for a spina bifida patient and the carers. But, hydronephrosis with progressive deterioration of renal function can occur insidiously. Urine infections may predispose to pyonephrosis. Such complications can be life-threatening. Therefore, every effort should be made to carry out intermittent catheterisations in persons with neuropathic bladder, who should also be prescribed antimuscarinic drugs. Successful implementation of intermittent catheterisation regime in spinal cord injury patients requires (1) education of the patient, (2) availability of carers, who have been trained to perform urethral catheterisation and (3) facilities for catheterisation within the house as well as in public places.

## **Consent**

This patient expired; therefore consent for publication of this case report was obtained from the next of kin of the deceased.

## **Competing interests**

The authors wish to state that the article processing fee for this manuscript will be paid by Hollister Limited, Rectory Court, 42 Broad Street, Wokingham, Berkshire, RG40 1AB, United Kingdom.

## **Authors' contributions**

SV conceived the idea and wrote the manuscript. PH reported medical images. FS was the consultant in charge of the patient. All authors participated in providing care to this patient. All authors read and approved the final manuscript.
